# Caution with colour calculations: spectral purity is a poor descriptor of flower colour visibility

**DOI:** 10.1093/aob/mcac069

**Published:** 2022-06-21

**Authors:** Casper J van der Kooi, Johannes Spaethe

**Affiliations:** Groningen Institute for Evolutionary Life Sciences, University of Groningen, The Netherlands; Department of Behavioral Physiology and Sociobiology, University of Würzburg, Germany

**Keywords:** Spectral purity, vision model, colour contrast, pollination, flower colour, saturation, chroma, plant–pollinator signalling

## Abstract

**Background:**

The colours of flowers are of key interest to plant and pollination biologists. An increasing number of studies have investigated the importance of saturation of flower colours (often called ‘spectral purity’ or ‘chroma’) for visibility to pollinators, but the conceptual, physiological and behavioural foundations for these metrics as well as the calculations used rest on slender foundations.

**Methods:**

We discuss the caveats of colour attributes that are derived from human perception, and in particular spectral purity and chroma, as variables in flower colour analysis. We re-analysed seven published datasets encompassing 774 measured reflectance spectra to test for correlations between colour contrast, spectral purity and chroma.

**Main findings and Conclusions:**

We identify several concerns with common calculation procedures in animal colour spaces. Studies on animal colour vision provide no ground to assume that any pollinator perceives (or responds to) saturation, chroma or spectral purity in the way humans do. A re-analysis of published datasets revealed that values for colour contrast between flowers and their background are highly correlated with measures for spectral purity and chroma, which invalidates treating these factors as independent variables as is currently commonplace. Strikingly, spectral purity and chroma – both of which are metrics for saturation and are often used synonymously – are not correlated at all. We conclude that alternative, behaviourally validated metrics for the visibility of flowers to pollinators, such as colour contrast and achromatic contrast, are better in understanding the role of flower colour in plant–pollinator signalling.

## INTRODUCTION

Flower coloration aids in attracting pollinators, which are vital for plant reproduction. As with any coloured object, the colours of flowers have different dimensions recognized by humans: hue, saturation (or chroma) and brightness (or lightness; for definitions of colour attributes see Table 1) (Kelber and Osorio 2010; Kemp *et al.*, 2015). Hue is the attribute or categorical description of an object’s shade, e.g. blue, green or red, and generally is the everyday meaning of ‘colour’. Saturation is the colourfulness of a stimulus relative to its own brightness, and chroma is colourfulness relative to the brightness of a similarly illuminated white area. For example, to humans red is more saturated than pink. Brightness and lightness are subjective measures of an object’s perceived absolute intensity or relative intensity as compared to a white area of similar intensity, respectively. The colours of flowers are caused by two different optical principles: absorption of light by floral pigments and reflection of light by scattering structures. The type of floral pigment determines a flower’s overall hue (van der Kooi *et al.*, 2016), and the amount of pigment roughly determines the chroma or saturation of flowers as perceived by humans (van der Kooi, 2021). The amount of reflected light (important for the perceived brightness) is principally determined by a flower’s backscattering structures. The above-mentioned dimensions of colours are defined based on human vision, yet are often (loosely) applied to pollinators, which is problematic because animal and human vision are markedly different (for discussion see below).

**Table 1.** Glossary of terms commonly used in flower colour and pollination literature, and the related optical mechanisms

Term	Definition	Optical mechanism in flowers
Colour	A visual perceptual property, primarily determined by the spectral composition of the perceived light, and the spectral sensitivities and neural processing of the observer.	Reflection of light occurs on the flower surfaces and irregularly structured inner flower components (e.g. vacuoles, air spaces and starch granules); wavelength-selective absorption by floral pigments causes the spectral modulation of the reflected light.
Colour contrast	Perceptual contrast in colour appearance of two objects (e.g. a flower and surrounding vegetation).	Primarily determined by the type of pigment of the flower and background.^1^
Achromatic (green) contrast	Perceptual contrast of two objects determined by the long-wavelength (green) photoreceptors only.	Determined by the amount of light-scattering structures and type of pigment of the flower and background.^1^
Hue	The categorical description of a colour, e.g. blue or red.	Primarily determined by the type of pigment(s).
Saturation	The colourfulness of an object relative to its own brightness.	NA – should only be applied to luminous light sources.
Chroma	The colourfulness of an object relative to a similarly illuminated white area.^2^	Primarily determined by the amount of pigment.
Spectral purity	In pollination literature considered as an object’s relative similarity to a monochromatic light of the same hue.	NA – not a formal dimension and should not be used as such (see main text).
Lightness	Perceived brightness of an object relative to a white object.	Primarily determined by the amount of light-scattering structures.
Brightness	Perceived intensity of a stimulus, independent of hue and saturation. Generally assumed to not be important for visibility to pollinators.	Primarily determined by the type and amount of light scattering structures.

^1^For calculations and applied colour spaces, see Chittka (1992), Chittka and Kevan (2005) or appendix in Kelber *et al* (2003). Colour contrast and achromatic contrast can be fairly straightforwardly calculated using various R packages (e.g. Maia *et al.*, 2013; Gawryszewski, 2018) or other software (e.g. Avicol; Gomez, 2006).

^2^The formal definition differs from how it is commonly used in pollination studies (see main text).

**Fig. 1.**
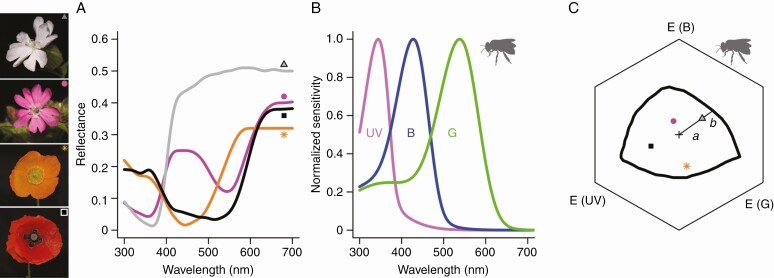
Examples of flower colours, bee spectral sensitivity and calculations for spectral purity. (A) Four example spectra for different flower colours. White flowers of *Silene latifolia-alba* (grey triangle), purple flowers of *Silene dioica* (magenta circle), yellow flowers of *Meconopsis cambrica* (orange star) and ultraviolet–red flowers of (European) *Papaver rhoeas* (black square). (B) Spectral sensitivity of honeybee photoreceptors, ultraviolet (UV); blue (B); green (G). (C) Honeybee hexagon plot of the four exemplary cases in panel A with corresponding symbols. The plotted monochromatic line is after Chittka (1992). Spectral purity for *S. latifolia-alba* is calculated as *a*/(*a* + *b*), where ‘*a*’ represents the flower’s colour contrast to the background. Different corners of the hexagon (marked with “E”) represent relative excitation of the different photoreceptors. The achromatic centre of the hexagon is indicated with a ‘+’ and is the background colour to which the bee photoreceptors are adapted. Honeybee silhouettes are from phylopic.org.

The colours of flowers are best interpreted when considering the visual perception of their pollinators. The visual contrast between a flower and its environment is a key factor for a flower’s visibility to pollinators (e.g. Spaethe *et al.*, 2001). Contrast can be between a flower and its (green leaf) background or between different flower parts, such as within-flower differences in ultraviolet (e.g. a ‘bulls eye pattern’). Two different types of contrast are important: colour contrast and achromatic contrast. Colour contrast is determined by the spectral differences (i.e. hue and saturation/chroma) between a flower and its surrounding, and is of great importance in object detection and recognition for numerous pollinator groups, including bees, butterflies, moths and vertebrates (Giurfa *et al.*, 1996; Kelber *et al.*, 2003; Dyer and Chittka, 2004; van der Kooi *et al.*, 2019). The spectral differences between a flower and its surrounding are largely caused by differences in absorption by pigments. Achromatic contrast, also known as green contrast, is a (colour-blind) contrast between stimulus and background, which is perceived by bees and presumably other insects by the long-wavelength (green) photoreceptors (Giurfa *et al*., 1996, 1997; Spaethe *et al.*, 2001; Goyret and Kelber, 2012; for a review, see van der Kooi and Kelber, 2022). Achromatic contrast is caused by both differences in absorption by pigments as well as light-scattering structures (Chittka *et al.*, 1994; Kevan *et al.*, 1996; van der Kooi and Kelber, 2022). Observer-subjective contrast is commonly calculated using vision models, e.g. the colour hexagon for trichromats (Fig. 1; see also Chittka, 1992) or the receptor noise-limited model (Vorobyev and Osorio, 1998). With easy access to software (e.g. the R package *Pavo*, Maia *et al.*, 2013) and a growing body of literature on pollinator spectral sensitivity (for reviews, see Briscoe and Chittka, 2001; van der Kooi *et al.*, 2021), it has become increasingly popular to analyse flower colour using vision models.

Inspired by early experiments by Lunau (1990, 1992, 1996), a growing number of studies are focusing on a measure of saturation of flower colours (often referred to as ‘spectral purity’ or ‘chroma’) as a key factor for flower visibility to bees (e.g. Rohde *et al.*, 2013; Shrestha *et al.*, 2014; Bergamo *et al.*, 2016; Koethe *et al*., 2016, 2018; Reverté *et al.*, 2016; Kantsa *et al.*, 2017; de Camargo *et al.*, 2019; Aguiar *et al.*, 2020; Chen *et al.*, 2020; Coimbra *et al.*, 2020; Howard *et al.*, 2021). Whereas the contrast values calculated with vision models generally scale well with conspicuousness to bees as well as other vertebrate and invertebrate pollinators (Chittka, 1992; Vorobyev and Osorio, 1998; Spaethe *et al.*, 2001; Kelber *et al.*, 2003; Dyer and Chittka, 2004; Kemp *et al.*, 2015; Renoult *et al.*, 2017), the evidence regarding spectral purity and chroma is mixed – even within bee species (see table 2 in van der Kooi *et al.*, 2019). Furthermore, the theoretical concept, calculation procedure and ecological significance of spectral purity and chroma in plant–pollinator signalling rest on slim foundations.

Here, we evaluate the theoretical framework, common calculation procedures and behavioural evidence of spectral purity and chroma of flowers as predictors for visibility to bee pollinators. We discuss the theory and empirical data on pollinator vision and colour processing, identify concerns with widely used calculation procedures, and argue that there is no behavioural evidence suggesting a function for spectral purity or chroma in bee vision. A re-analysis of seven published datasets encompassing 774 measured reflectance spectra revealed that spectral purity and chroma are highly correlated with colour contrast and therefore should not be used as an independent metric for conspicuousness in plant–pollinator signalling.

## WHAT DO BEHAVIOURAL AND PHYSIOLOGICAL SCIENCES TELL US ABOUT THE PERCEPTION OF SATURATION?

The psychology of colour perception in humans is complex and several colour appearance models have been developed to explain different aspects. According to the Commission Internationale de l’Eclairage (CIE) a colour appearance is assigned several attributes such as hue, colourfulness, brightness, lightness, chroma and saturation, depending on the colour model (Fairchild, 2013). The terms saturation and chroma are often used synonymously in pollination literature, despite their different meanings and definitions by the CIE. Chroma refers to the perceived colour difference between a stimulus and a white area of similar brightness. Chroma is applicable only to non-luminous stimuli (e.g. coloured paper, fabric and paint) (Hunt, 1978). Saturation refers to the colourfulness of a stimulus relative to its own brightness and is primarily used for luminous stimuli (e.g. coloured lights, LEDs). In other words, to estimate the chroma of a stimulus it must be judged in relation to other colours, whereas a stimulus seen in complete isolation can have saturation but no chroma (Fairchild, 2013). For example, a traffic light at night viewed against a black sky exhibits saturation but no chroma.

Whether these colour attributes are also perceived by animals other than humans is largely unknown (Kelber and Osorio, 2010). Honeybees, for example, largely ignore brightness when they discriminate coloured lights (Daumer, 1956; Menzel, 1967; Backhaus, 1992), which makes it doubtful whether they perceive saturation in the way humans do. An important missing piece of evidence to interpret the importance of saturation and chroma for visibility to pollinators is that there is no convincing behavioural data that describe the response of bees or any other insect to stimuli that vary only in saturation. Furthermore, for flowers with yellow, orange and red colours there is the additional concern that they often also reflect light in the ultraviolet wavelength range. Flowers that solely reflect yellow light are of higher chroma than stimuli that also reflect ultraviolet light (Lunau, 1992), but for ultraviolet-sensitive insects these two stimuli also have different hues.

## CONCERNS WITH CALCULATIONS FOR SPECTRAL PURITY AND CHROMA

The spectral purity and chroma of flowers are commonly calculated via two different approaches. Spectral purity, which of the two is most commonly used in pollination studies, is calculated using Chittka’s hexagon model (note that the brightness or lightness dimension is ignored in all insect vision models) (Chittka, 1992). The hexagon colour space is enclosed by a line that represents the loci of monochromatic stimuli, which are, by definition, colours of maximum chroma/saturation. The spectral purity of a stimulus is calculated by taking the distance between the central, achromatic point and the stimulus, and dividing that by the distance between the central point and the monochromatic locus of the specific hue (Lunau, 1990; Chittka and Kevan, 2005; Rohde *et al.*, 2013). For example, for the white flowers of *Silene latifolia-alba*, spectral purity would be *a*/(*a* + *b*), with ‘*a*’ being the colour contrast between the flower and background (Fig. 1C).

The receptor noise-limited (RNL) model provides no similar approach to calculate chroma/saturation. In studies where the RNL model was used to calculate the colour contrast of flowers to the background, chroma was calculated in the same way as spectral purity (thus using the hexagon vision model) or via an approach independent from vision models, meaning it was considered a strictly physical property. In that vision model-independent approach, ‘chroma’ is obtained by subtracting the maximal and minimal reflectance value, and dividing that by the average reflectance (Endler, 1990; Valido *et al.*, 2011). That approach was, to the best of our knowledge, first described by Endler (1990), though studies in pollination biology often cite Valido *et al.* (2011). Although the obtained value is commonly called ‘chroma’, this is technically incorrect wording because ‘true’ chroma is a percept relative to the brightness of the stimulus (see the previous section: ‘What do behavioural and physiological sciences tell us about the perception of saturation?’, and Table 1). Brightness, however, is not a relevant element in insect vision, so it is not incorporated in insect vision models.

Spectral purity and ‘chroma’ are calculated and interpreted in a linear way; however, how these linear metrics scale with pollinator behavioural responses is untested. For example, a change in spectral purity or ‘chroma’ from 10 to 20 % is treated with the same weight as an increase from 60 to 70 %, but psychophysical and behavioural responses are rarely linear in nature. Indeed, behavioural responses to visual stimuli often follow a sigmoidal response curve (Olsson *et al.*, 2015; Garcia *et al.*, 2017; Santiago *et al.*, 2020). It would be interesting to know if insects respond linearly to stimuli with varying degrees of spectral purity or ‘chroma’, as has been investigated with chickens (Scholtyssek *et al.*, 2016).

**Fig. 2.**
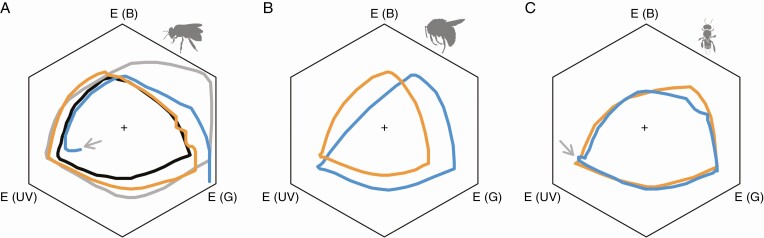
Examples of monochromatic lines for different species and from different studies. (A) Honeybees, black line (Chittka 1992), blue line (Dyer and Neumeyer 2005), grey line (Chittka *et al.* 1994) and orange line (Dafni *et al.* 2020). (B) Bumblebees, orange line (Bergamo *et al.* 2016), blue line (Papiorek *et al.* 2013). (C) Stingless bees, blue line (Chittka *et al.* 1992) and orange line (Koethe *et al.* 2016). Different corners of the hexagon (marked with “E”) represent relative excitation of the different photoreceptors. The achromatic centre of the hexagon is indicated with a ‘+’ and is the background colour to which the bee photoreceptors are adapted. Arrows in A and C highlight artefacts that arise in the ultraviolet wavelength range (see ‘Concerns with calculations for spectral purity and chroma’). Bee silhouettes are from phylopic.org.

The monochromatic line that forms the boundary of a bee’s colour space is a key factor in calculating spectral purity. To visualize the colour spaces for different bee species, we extracted the monochromatic lines from several publications for honeybees (*Apis mellifera*), bumblebees (*Bombus terrestris*) and stingless bees (*Melipona quadrifasciata*) and plotted them per species (Fig. 2). There exists striking variation in the shape and location of the monochromatic lines between studies, even for the same model species. Although the original works frequently used the same calculation procedure and source for spectral sensitivities (e.g. Peitsch *et al.*, 1992; Briscoe and Chittka, 2001), in almost all cases the monochromatic lines do not overlap. It is not clear why there is so much variation between studies because the underlying data and code are unavailable for these studies, but we can conclude that the large variation will greatly impact the value for spectral purity. The differences in where the monochromatic line is located are largest in the top right corner of the hexagon, where white and yellow stimuli are located, which is also the part where generally most flower species are plotted (Chittka *et al.*, 1994). For our exemplary case of *S. latifolia-alba*, there is a two-fold difference in distance from the centre to the monochromatic line depending on which monochromatic line is used (compare grey vs. black/orange in Fig. 2A).

There are several salient concerns pertaining to calculations of the monochromatic line, which may explain why there is so much variation between studies. First, drawing a monochromatic line between 700 and 300 nm is problematic. Whereas the line from 300 to 700 nm (the lower border) can be calculated using values for (existing) monochromatic stimuli in the ultraviolet, blue, green, yellow and red wavelength ranges, there is no equivalent for the range between 700 and 300 nm, because such stimuli do not exist. Indeed, the electromagnetic (light) spectrum is linear and not circular. The common way to circumvent this problem is that stepwise fractions of the values at 700 and 300 nm are used in the calculation (Chittka and Kevan, 2005; Rohde *et al.*, 2013). Although this may be a convenient way to obtain a lower border of the colour space, there is no fundamental justification for this procedure and the resulting lower border thus is hypothetical. The values of 300 and 700 nm also appear subjective and chosen out of convenience, because they could as well have been 320 or 280, or 680 or 720.

Second, between 300 and 350 nm the monochromatic line in the hexagon sometimes features sharp turns or a hook towards the achromatic point (arrows in Fig. 2A, C). Those abrupt changes are artefacts caused by the fact that not only the UV-photoreceptor but also the blue and green photoreceptors have (low) sensitivity in the ultraviolet (Fig. 1B), which is the so-called beta-peak (e.g. Stavenga *et al.*, 1993; Govardovskii *et al.*, 2000; Stavenga, 2010). When calculating the monochromatic loci between 300 and 350 nm, all photoreceptors are excited. Close to 300 nm the excitation difference between the photoreceptors becomes comparatively small, so the loci are pulled towards the achromatic centre. Some studies avoid this problem by stopping the line at 350 nm, and then connect it to the 700-nm point using the stepwise fraction, although there is no fundamental justification for that procedure.

Third, for several species and various parts of the colour space, the overall distance between the centre point and the monochromatic line varies little. Except for the cases where the monochromatic line almost borders the edges of the hexagon (e.g. the grey curve in Fig. 2A), for most colour spaces the distance between the centre and the monochromatic line is fairly constant. Not only does this increase the bias caused by artefacts because more weight is given to very small variation, it also presents a problem for calculating spectral purity. Given that the distance between the centre point and the monochromatic line is the denominator in the calculation of spectral purity, with decreasing variation in the denominator, the numerator will be of increasing importance. In calculating spectral purity, the numerator is the distance between the centre point and the stimulus (i.e. colour contrast), so we can expect spectral purity to correlate strongly with colour contrast.

## SPECTRAL PURITY AND COLOUR CONTRAST ARE HIGHLY CORRELATED

To interpret the importance of different metrics for a behavioural response, it requires those metrics to be independent. Complete independence of different colour metrics is virtually impossible, because the reflectance at a given wavelength is strongly dependent on reflectance at neighbouring wavelengths. Nevertheless, because reflectance spectra are modulated over a roughly 400-nm wavelength range (between 300 and 700 nm) and sensitivity of different photoreceptors varies greatly, some largely independent metrics can be calculated. Indeed, colour contrast and (achromatic) green contrast are not necessarily correlated (Garcia *et al.*, 2021).

To test for possible correlations between values for chroma/spectral purity and colour contrast, we re-analysed results from previously published papers. Excluding studies based on fewer than 15 stimuli (e.g. Gumbert, 2000; Papiorek *et al.*, 2013; Rohde *et al.*, 2013; Dyer *et al.*, 2016; Howard *et al.*, 2021) and those for which underlying data were unavailable, we obtained information from seven published studies, in total covering 774 measured reflectance spectra (Table 2). Values for spectral purity/chroma and colour contrast between the flower and background were obtained for flowers of different species (most studies), for different colour morphs (Bergamo *et al.*, 2016) or for artificial flowers that were used in behavioural assays (two studies; Table 2). The stimuli were analysed using honeybee, bumblebee and/or stingless bee visual systems (see Table 2 for details on the datasets analysed). We tested for correlations using Spearman’s rank correlation. Reported *P*-values are Benjamini–Hochberg corrected for multiple testing.

**Table 2.** Studies used in our analysis, the applied visual system and vision model, and the way(s) to calculate ‘saturation’. ‘Saturation’ was calculated using the relative distance to the monochromatic point in the hexagon (‘spectral purity’) or using a vision model-independent approach based on the measured reflectance spectrum (‘chroma’; as per Endler, 1990)

Dataset	Number and type of stimuli analysed	Visual system	Chromatic contrast calculation	Saturation calculation
Papiorek *et al*. (2014)	58 species, 2 floral structures per species	Honeybee	Hexagon and RNL	Spectral purity and ‘chroma’
Bergamo *et al*. (2016)	30 white/pink morphs of *Costus arabicus*, 2 floral elements per individual	Bumblebee	RNL	Spectral purity
Shrestha *et al*. (2014)	105 plant species	Bumblebee^1^	Hexagon	Spectral purity
Koethe *et al*. (2016)	16 artificial stimuli of 4 colour categories	*Melipona quadrafasciata*	Hexagon	Spectral purity
Ortiz *et al*. (2021)	98 plant species	Honeybee	Hexagon	‘Chroma’
Koethe *et al*. (2018)	20 artificial stimuli	*Melipona quadrafasciata*	NA	Spectral purity and ‘chroma’
Coimbra *et al*. (2020)	389 species	Honeybee, bumblebee	Hexagon	Spectral purity

^1^The specific species was not given in the original publication, but the used spectral sensitivities are similar to those of the bumblebee.

**Fig. 3.**
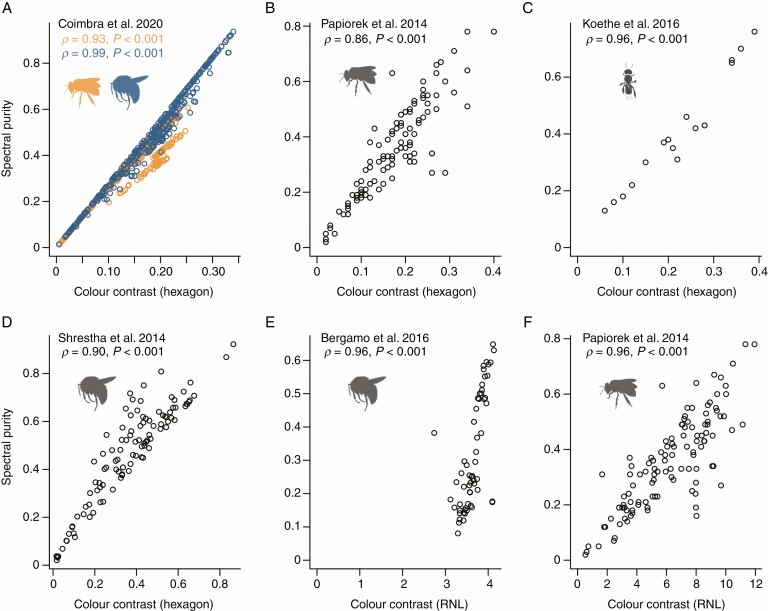
Spectral purity and colour contrast between flower and background calculated using the hexagon are strongly correlated (A–D). Colour contrast as per the receptor noise-limited (RNL) model and spectral purity as per the hexagon approach are also strongly correlated (E, F). The largest dataset (A) provided data for both honeybees (orange) and bumblebees (blue). The unusual distribution in panel E is because that study included two colour morphs of one species. Details regarding the analysed datasets are given in Table 2. Bee silhouettes are from phylopic.org.

Our analysis showed that colour contrast and spectral purity are highly correlated, for honeybees, bumblebees and stingless bees (Fig. 3). All five analyses using the hexagon model to calculate both colour contrast and spectral purity yielded highly significant correlations with an extremely high correlation coefficient (ρ: 0.86–0.99, *P* < 0.001; Fig. 3A–D). Similarly, when comparing colour contrast as per the RNL model and spectral purity as per the hexagon model, both are highly correlated (ρ = 0.96, *P* < 0.001 for both datasets), which is in line with previous studies that reported that the hexagon and RNL models often yield comparable results (Dyer and Neumeyer, 2005; van der Kooi *et al.*, 2016; Gawryszewski, 2018).

**Fig. 4.**
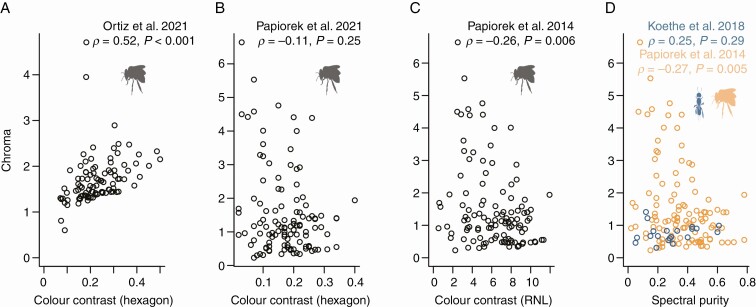
‘Chroma’ can be positively correlated, uncorrelated or negatively correlated with colour contrast (A-C). Spectral purity and ‘chroma’ are not (positively) correlated (D). Bee silhouettes are from phylopic.org.

The correlation between spectral purity and colour contrast has been pointed out before (e.g. Aguiar *et al.*, 2020; Coimbra *et al.*, 2020; Howard *et al.*, 2021), though the authors of these studies then included only spectral purity in their subsequent analyses (Aguiar *et al.*, 2020; Howard *et al.*, 2021) or actually concluded that because of the strong correlation it is meaningful to include both spectral purity and colour contrast in subsequent analyses (Coimbra *et al.*, 2020). We argue that because the effect of spectral purity cannot be disentangled from the effect of colour contrast – both statistically (Figs 3 and 4) and conceptually (see above) – one should use colour contrast, for which the calculation is least ambiguous and for which clear behavioural evidence supporting its relevance for visibility to pollinators exists.

## ‘CHROMA’ IS AN UNRELIABLE METRIC OF FLOWER VISIBILITY

When plotting colour contrast against the commonly used vision model-independent metric for ‘chroma’ (*sensu*Endler, 1990; Valido *et al.*, 2011), the results are mixed. In the largest of the two available datasets (Ortiz *et al.*, 2021) hexagon colour contrast is significantly correlated with chroma (ρ = 0.52, *P* < 0.001; Fig. 4A). RNL colour contrast negatively correlates with chroma (ρ = −0.26, *P* = 0.006), though this could be tested for only one dataset (Papiorek *et al.*, 2014; Fig. 4C).

Another striking observation is that spectral purity and ‘chroma’ – which are commonly used synonymously and would be expected to be correlated – actually are virtually uncorrelated. Two studies calculated both spectral purity using the hexagon approach and the (model-independent) chroma (Papiorek *et al.*, 2014; Koethe *et al.*, 2018), which enabled us to compare how these two metrics correlate. The comparatively small dataset of Koethe *et al.* (2018) (*n* = 20 stimuli) did not reveal a significant correlation. Counter-intuitively, the larger dataset of Papiorek *et al.* (2014) suggested that, if anything, the two metrics are actually *negatively* correlated (ρ = −0.27, *P *= 0.005, Fig. 4D).

Another caveat of the commonly used metric for ‘chroma’ is that it is entirely based on the spectral reflectance of an object and ignores the visual system of the observer. Consequently, the chroma, for example, of a yellow flower that also reflects ultraviolet light is the same for ultraviolet-sensitive bees and ultraviolet-insensitive humans, although both perceive a completely different colour. The value for ‘chroma’ also depends on the wavelength range used for the calculation. Flowers and leaves, for example, significantly reflect light in the near-infrared above 700 nm (van der Kooi *et al.*, 2016) and so become more ‘chromatic’ when the used spectral range extends into longer wavelengths. Therefore, ‘chroma’ (*sensu*Endler, 1990; Valido *et al.*, 2011) is an inadequate indicator for how flower colour is perceived by pollinators.

## CONCLUDING REMARKS

We argue that the concept, theory and calculation of flower spectral purity and ‘chroma’ (*sensu*Endler 1990) are conceptually weak and should not be used to describe flower visibility to pollinators. Analyses of published datasets revealed that the common way to calculate spectral purity (calculated as the relative distances to monochromatic stimuli) yields values that are extremely correlated with values for colour contrast between the flower and background. Such extreme correlations of two variables means that they should not be treated, as often happens, as two independent variables. Including an extra variable further increases the chance of introducing false positives, particularly in studies where many different flowers are analysed. The two different metrics for saturation – spectral purity and ‘chroma’ – are further entirely uncorrelated, presumably because ‘chroma’ is calculated using the reflectance spectrum, independent from the visual system. Finally, the monochromatic line that constitutes the boundary of a pollinator’s colour space serves no purpose for any calculations, and should only be used to illustrate the potential breadth of and different regions in the colour space that can be filled with (flower) colours. That is the case for the hexagon as well as other vision models, such as a colour triangle or tetrahedron for tetrachromats.

If colour contrast between flower and background is *de facto* the same as spectral purity, why then not use either one of the two? We argue that colour contrast is a better metric than spectral purity (and ‘chroma’) for several reasons: (1) the ecological importance of colour contrast is broadly supported by behavioural and physiological data; (2) colour contrast is a less anthropocentric and so more objective way of interpreting the perception of visual signals; (3) whereas spectral purity and ‘chroma’ can only be calculated per flower, differences in colour contrast can also be calculated between two stimuli, such as two flowers or differently coloured flower parts; and (4) perhaps most importantly: keep it simple. After Occam’s razor: ‘lex parsimoniae’, i.e. entities should not be multiplied beyond necessity. The calculation procedure for colour contrast is superior to spectral purity because it relies on fewer calculations and assumptions. As long as the explanatory value of spectral purity does not exceed that of colour contrast and there is no behavioural evidence supporting its validity, there is no scientific basis for its usage. Implementing behaviourally validated sigmoidal response curves, i.e. functions that describe how (step-wise) changes in perceived colour contrast change the behavioural response of the observer (Olsson *et al.*, 2015; Garcia *et al*., 2017, 2020; Santiago *et al.*, 2020), may enhance the ecological realism of colour contrast as a proxy for visibility. Achromatic contrast is another useful proxy for visibility of flowers by pollinators, particularly from long distances, and is straightforward to calculate (e.g. Spaethe *et al.*, 2001; van der Kooi and Kelber, 2022).

In addition to the conceptual problems regarding saturation, chroma and spectral purity, we observed many inconsistencies in terminology in the (pollination) literature. In some cases, the terms saturation, spectral purity, chroma and/or colour contrast seem to be used synonymously (e.g. Chittka *et al.*, 1994; Lunau *et al.*, 1996; Chittka and Wells, 2004; Maia *et al.*, 2013), despite their different definitions. For example, in manuals and online examples of the popular software tool *Pavo* (e.g. versions 0.5.1 and 2.0.0; see also Maia *et al.*, 2013), we read that the variable that represents the distance from the centre to the stimulus (i.e. colour contrast between the flower and background), the ‘r.vec’ value, is called ‘saturation’. We could not always deduce how ‘saturation’ was calculated in some publications, but in at least one study (i.e. Gray *et al.*, 2018) the ‘r.vec’ output was taken as a measure for saturation. Similar mixing of terminology occurs in the tetrahedral colour space of Stoddard and Prum (2008) that is popular to model bird vision, where ‘saturation’ is also calculated as the distance from the achromatic centre to the stimulus. Although this need not be a problem when the methods and calculations are clearly reported, inadequate usage of terminology and processes frequently leads to inaccuracies and confusion in the literature. What may have fuelled inaccurate use of terminology as well as the perpetuation of the whole concept of flower colour saturation is the slightly mystical connotation of the term ‘spectral purity’.

We wish to emphasize that we are not principally against using the terms and concepts of hue, chroma/saturation or brightness/lightness – they can indeed be useful to characterize the spectral properties of objects. However, chroma and saturation are colour attributes defined for human colour perception, and in the current absence of quality pollinator psychophysics data they should not be used in studies on flower colour. Instead, we suggest to keep it simple and use metrics that are corroborated by physiological and behavioural data. Colour and achromatic contrast are well-established measures that provide species-specific behaviourally and physiologically meaningful measures for flower conspicuousness.
